# Association of gut microbiota with sort-chain fatty acids and inflammatory cytokines in diabetic patients with cognitive impairment: A cross-sectional, non-controlled study

**DOI:** 10.3389/fnut.2022.930626

**Published:** 2022-07-22

**Authors:** Yage Du, Xiaoying Li, Yu An, Ying Song, Yanhui Lu

**Affiliations:** ^1^School of Nursing, Peking University, Beijing, China; ^2^Geriatrics Department, Beijing Jishuitan Hospital, Beijing, China; ^3^Endocrinology Department, Beijing Chaoyang Hospital, Beijing, China

**Keywords:** type 2 diabetes mellitus, mild cognitive impairment, gut microbiota, 16S rRNA gene, short-chain fatty acids, inflammatory cytokines

## Abstract

Emerging evidence suggests that gut microbiota, short-chain fatty acids (SCFAs), and inflammatory cytokines play important roles in the pathogenesis of diabetic cognitive impairment (DCI). However, little is known about alterations of gut microbiota and SCFA levels as well as the relationships between inflammatory cytokines and cognitive function in Chinese DCI patients. Herein, the differences in the gut microbiota, plasma SCFAs, and inflammatory cytokines in DCI patients and type 2 diabetes mellitus (T2DM) patients were explored. A cross-sectional study of 30 DCI patients and 30 T2DM patients without mild cognitive impairment (MCI) was conducted in Tianjin city, China. The gut microbiota, plasma SCFAs, and inflammatory cytokines were determined using 16S ribosomal RNA (rRNA) gene sequencing, gas chromatography-mass spectrometry (GC-MS), and Luminex immunofluorescence assays, respectively. In addition, the correlation between gut microbiota and DCI clinical characteristics, SCFAs, and inflammatory cytokines was investigated. According to the results, at the genus level, DCI patients presented a greater abundance of *Gemmiger, Bacteroides, Roseburia, Prevotella*, and *Bifidobacterium* and a poorer abundance of *Escherichia* and *Akkermansia* than T2DM patients. The plasma concentrations of acetic acid, propionic acid, isobutyric acid, and butyric acid plummeted in DCI patients compared to those in T2DM patients. TNF-α and IL-8 concentrations in plasma were significantly higher in DCI patients than in T2DM patients. Moreover, the concentrations of acetic acid, propionic acid, butyric acid, and isovaleric acid in plasma were negatively correlated with TNF-α, while those of acetic acid and butyric acid were negatively correlated with IL-8. Furthermore, the abundance of the genus *Alloprevotella* was negatively correlated with butyric acid, while that of *Holdemanella* was negatively correlated with propanoic acid and isobutyric acid. *Fusobacterium* abundance was negatively correlated with propanoic acid. *Clostridium XlVb* abundance was negatively correlated with TNF-α, while *Shuttleworthia* abundance was positively correlated with TNF-α. It was demonstrated that the gut microbiota alterations were accompanied by a change in SCFAs and inflammatory cytokines in DCI in Chinese patients, potentially causing DCI development. These findings might help to identify more effective microbiota-based therapies for DCI in the future.

## Introduction

Diabetes mellitus (DM) has been widely acknowledged as a major global health threat. The International Diabetes Federation (IDF) estimated that the number of DM adults (aged from 20 to 79) increased from 285 million in 2009 to 463 million in 2019, with type 2 diabetes mellitus (T2DM) patients accounting for 95% of the total (1). T2DM generally induces cognitive decline, manifested as mild to moderate cognitive impairment, lessened learning and memory capability, and weakened executive ability (2–4). Mild cognitive impairment (MCI) is a modifiable stage between normal cognitive aging and dementia (5), prevalent in T2DM patients. The combined prevalence of MCI and T2DM was estimated to be as high as 45.0% (95% CI = 36.0–54.0%), as reported by a recent meta-analysis covering 5015 T2DM cases (6). The China Longitudinal Aging Study (CLAS) identified that MCI prevalence in T2DM patients was 21.8% in China (2), while another study showed that the figure reached up to 28% in Tianjin city (3). This underscores the importance of drawing attention to the higher risk of cognitive impairment in diabetic patients than in non-diabetic patients (7). T2DM, combined with neurocognitive dysregulation, contributes to the progression of Alzheimer's disease (AD) and severely lower life quality in older adults (8). Additionally, the etiology and effective treatments of diabetic cognitive impairment (DCI) still remain uncertain, despite extensive research over the past few decades. In this sense, the early detection of DCI is critical for preventing cognitive impairment progression in T2DM patients.

The gut microbiota comprises trillions of symbiotic microorganisms, whose alterations impact not only gut diseases but also central nervous system (CNS) disorders like DCI (9). Gut microbiota induces amyloid-beta (Aβ) aggregation, neural injury, insulin resistance, oxidative stress, mitochondrial dysfunction, synaptic disorder, and neuroinflammation in the pathogenesis of DCI, ultimately causing neurodegeneration (10–12). The term microbiota-gut-brain axis (MGBA) was coined to describe the gut microbiota-brain. Substantial evidence revealed the linkage between DCI pathogenesis and microbial dysbiosis (13, 14). DCI patients represented a decreased relative abundance of *Tenericutes, Bifidobacterium*, and *unranked-RF39* but a significantly increased abundance of *Peptococcus* and *unranked-Leuconostocaceae* (15). An animal experiment identified the association between the abnormal gut microbial composition with cognitive dysfunction in diabetic mice (16). In AD and DCI model mice, gavage of *Akkermansia muciniphila* (Akk) could delay pathological changes in the brain and alleviate spatial learning memory impairment (17, 18), suggesting that intervention of gut microbiota might be a promising therapeutic approach for DCI. However, how the gut microbiota contributes to the pathogenesis and progression of DCI is still unclear.

Inflammation significantly exacerbated DCI by increasing Aβ accumulation, tau hyperphosphorylation, and neuronal cell death (19–22). Remarkably, the gut microbiota alterations promoted immune activation and the blood-brain barrier (BBB) (11, 23). For this reason, neurotoxic bacteria, cytokines, and chemokines could induce neuronal structure change, neuronal cell death in the brain through BBB, and eventually cognitive function (24, 25). Inflammatory cytokines had close ties to DCI, as reported by a recent study that serum high-sensitivity C-reactive protein (hs-CRP) and pro-inflammatory cytokines such as interleukin-6 (IL-6) and tumor necrosis factor-α (TNF-α) were elevated in DCI patients (21). Moreover, an animal study revealed that inflammatory genes were significantly up-regulated in the brain tissue of DM mice (26). In addition, T2DM could cause microglial activation and subsequent production of pro-inflammatory cytokines in db/db mice (14). Dendrobium mixture has been shown to improve the cognitive and memory function by restoring the homoeostasis of gut microbiota in DCI mice attributed to its anti-inflammatory activities (27). However, anti-inflammatory medicines failed to be used in clinical studies, and the underlying mechanism remained unclear (28).

Short-chain fatty acids (SCFAs), as saturated aliphatic organic acids containing 1-6 carbons, were reported to be also involved in DCI pathogenesis. Ninety percent of SCFAs in the human body were bacterial fermentation products of fiber (29). Microbiota-derived SCFAs were key mediators for the gut-brain axis and relevant to Aβ plaque deposition (30, 31). SCFAs also influenced CNS function in various ways, including changes in neurotransmitter production, mitochondrial function, immune activation, lipid metabolism, gene expression, and neuronal function (32). Results of a recent study indicated that long-term acetic acid deficiency resulted in an impaired learning and memory in type 1 diabetes mellitus (T1D) mice. Exogenous acetate supplementation improved learning and memory impairments through raised hippocampus SYP levels, suggesting that acetic acid plays an important role in cognitive decline in mice (33). By increasing butyric acid levels, fecal microbiota transplantation (FMT) could alleviate AD-like pathogenesis in APP/PS1 transgenic mice (34). Butyric acid could also reduce cognitive dysfunction and enhance brain-derived neurotrophic factor (BDNF) expression. Moreover, SCFAs played an important role in AD by the gut microbiota through the inflammatory pathway. Specifically, SCFAs could induce microglial maturation and promote Aβ plaque seeding and growth in the early stages of AD pathology. The Aβ-activated microglia further produced neurotoxic cytokines and chemokines, including TNF-α, IL-6, IL-1, and CCL2, resulting in neuronal dysfunction and cell death (30, 35). However, very few studies revealed the gut microbiota alterations and SCFA levels in DCI patients, as well as the relationships between inflammatory cytokines and cognitive function in DCI patients.

Although the correlation between gut microbiota and DCI pathology has received much attention in the last decade, the underlying mechanism of DCI remains to be explored. To our knowledge, no study has investigated the correlation between gut microbiota and SCFAs or inflammatory cytokines in DCI patients in China. Therefore, in this paper, a cross-sectional study was conducted to examine and compare the gut microbiota communities in the feces of DCI and T2DM patients using 16S ribosomal RNA (rRNA) gene sequencing. Gas chromatography-mass spectrometry (GC-MS) and Luminex immunofluorescence assays were carried out to measure the plasma concentrations of SCFAs and inflammatory cytokines. Additionally, the correlation of gut microbiota with DCI clinical characteristics, SCFAs, and inflammatory cytokines was investigated.

## Materials and methods

### Subjects

This study complied with the Declaration of Helsinki and was approved by the Ethical Committee of the Health Science Center, Peking University (IRB00001052-21131). All participants agreed to take part in the investigation and signed an informed consent agreement before the study. They enjoyed the right to withdraw at any time during study. A total of 60 T2DM patients were recruited in Tianjin city, China (36) and divided into DCI group (30 patients with impaired cognition) and T2DM group (30 patients with normal cognition). The age, gender, and body mass index (BMI) of T2DM patients were matched with those of DCI patients. All the participants were aged 60 years and older. T2DM patients were diagnosed in accordance with the American Diabetes Association (ADA) criteria, i.e., HbA_1C_ ≥ 6.5%, FBG ≥ 126 mg/dL (7.0 mmol/L), or 2-h plasma glucose ≥ 200 mg/dL (11.1 mmol/L) during an OGTT (37). MCI patients were screened by the Beijing version of the Montreal Cognitive Assessment (MoCA-BJ) scales and received a diagnosis from an experienced neurologist. The MoCA-BJ score of below 26 was applied to MCI diagnosis (one point was added for those with <12-year education) (15, 38). DCI refers to MCI that occurs in patients with T2DM (39). The Chinese version of the Mini-Mental State Examination (MMSE) and Clock Drawing Test (CDT) were also employed to assess the cognitive function of participants. Participants were excluded if they: (1) were diagnosed with schizophrenia or other mental illness, (2) underwent acute surgery or serious infections recently, (3) took antibiotics or probiotics in the last 6 months, (4) had alcohol and substance abuse, or (5) presented the severe visual, hearing, and speaking impairments.

### Demographic characteristics and clinical data collection

Demographic characteristics and clinical data of all participants were collected through face-to-face interviews using a standard questionnaire. The height, weight, and waist circumference (WC) of subjects were measured, and the BMI was calculated by the formula of weight (kg) divided by the square of the height (m^2^). Blood pressure was determined using the professional portable blood pressure monitor (OMRON). Exercise frequency (daily, occasionally, and never), smoking, and alcohol consumption (frequently, occasionally, and never) were also assessed.

### Blood sample collection and detection

Blood samples (10 ml) were collected from each participant after a 10-h overnight fast. Subsequently, biochemical analyses were performed to measure the concentrations of fasting blood glucose (FBG), hemoglobin A1c (HbA_1_C), alanine aminotransferase (ALT), aspartate aminotransferase (AST), total bilirubin (TBIL), creatinine (Cr), blood urea nitrogen (BUN), total cholesterol (TC), triglyceride (TG), low-density lipoprotein-cholesterol (LDL-C), and high-density lipoprotein cholesterol (HDL-C) using the Mindray BS-350E blood biochemical analyzer (Shenzhen, China) at Peking University, Beijing, China.

### Measurement of plasma inflammatory cytokines

The plasma was isolated from blood samples and stored at−80°C before processing. Then it was used to detect the concentration of SCFAs and inflammatory cytokines. The plasma levels of TNF-α, IL-6, IL-8, IL-10, monocyte chemoattractant protein-1 (MCP-1), IL-1β, interferon-γ (IFN-γ), TNF-β, granulocyte-macrophage colony-stimulating factor (GM-CSF), and granulocyte colony-stimulating factor (G-CSF) in plasma were tested using Luminex immunofluorescence multiplex assays following the manufacturer's instructions.

### Measurement of plasma SCFAs

The concentrations of plasma SCFAs, including acetic acid, propionic acid, isobutyric acid, butyric acid, isovaleric acid, and valeric acid, were determined using the GC-MS spectrometer (Agilent 689N/5975B, USA). Phosphoric acid was used to deproteinize plasma samples, which were then extracted using ether. The supernatant was collected and injected into GC-MS after the centrifugation at 12,000 rpm for 10 min. An Agilent 689N/5975B GC-MS was fitted with a capillary column HP-INNOWAX (30 m × 0.25 mm ID × 0.25 μm), with an injection volume of 1 μL at 250 °C. The temperatures of the ion source and the transmission line were 230 and 250°C, respectively. The set carrier gas flow rate was 1.0 mL/min. The electron bombarding voltage was 70 eV, and the single ion monitoring was performed.

### DNA extraction, PCR, and 16S rRNA gene amplicon sequencing

A total of 10 g of fecal samples were collected from each participant in the morning using fecal collection containers and immediately transferred to−80°C. Afterward, the total fecal genomic DNA was extracted using the QIAamp Fast DNA Stool Mini Kit (Qiagen, Germany), following the manufacturer's instructions. In detail, each 200 mg of feces was mixed with 1 ml of InhibitEX buffer and a proper amount of glass beads (0.5 mm diameter, Qiagen), which was then homogenized and beat twice at 60 Hz for 1 min with a Homogeneous instrument (FASTPREP-24, Aosheng Biotech, China). Then, the DNA purification was performed following the manufacturer's instructions. NanoDrop 2000 (Thermo Scientific, USA) was used to control the quantity and purity of the extracted DNA. Agarose gel electrophoresis was performed to evaluate the integrity and size of the isolated DNA.

PCR was performed using the KAPA HiFi HotStart ReadyMixPCR Kit (KapaBiosystems, Boston, USA): 95°C for 3 min, 30 cycles at 98°C for 20 s, 58°C for 15 s, 72°C for 20 s and a final extension at 72°C for 5 min. PCR reactions occurred in a 30 μL of a mixture containing 15 μL of 2 × KAPA Library Amplification ReadyMix, 1 μL of each primer (10 μM), 50 ng of template DNA and ddH_2_O. Universal primers (341F: 5'-CCTACGGGRSGCAGCAG-3' and 806R: 5'-GGACTACVVGGGTATCTAATC-3') were employed to amplify the V3-V4 region of the 16S rRNA gene. Amplicons extracted from 2% of agarose gels were purified using the AxyPrep DNA Gel Extraction Kit (Axygen Biosciences, Union City, CA, USA) following the manufacturer's instructions and quantified using Qubit® 2.0 (Invitrogen, USA). Afterward, the quantified amplicons were sequenced using the Illumina MiSeq/HiSeq platform (San Diego, CA, USA). The paired-end reads of 250 bp were overlapped on their three ends for concatenation into the original longer tags using PANDAseq (https://github.com/neufeld/pandaseq, version 2.9). The DNA extraction, library construction and sequencing were conducted at the Realbio Genomics Institute (Shanghai, China) (40). Stripped of barcodes and primers, the assembled tags were tested in terms of the rest length and average base quality. The length of 16S tags was limited to 220-500 bp. The copy number of tags was enumerated, and the redundant duplicate tags were removed. Only the tags with a frequency of above 1 were clustered into operational taxonomic units (OTUs), with each unit having a representative tag. Sequences with more than 97% similarity were clustered to generate OTUs using Usearch software (version 7.0), with each OTU considered to represent one species (32). “Unclassified” tags were those incapable of being assigned to any known taxonomic level. The OTU profiling and α/β-diversity analyses were also assessed by python scripts of QIIME (version 1.9.1).

### Statistical analysis

SPSS (version 23.0) and R software (version 4.0.4) were employed for statistical analysis. The normality test was performed using the Shapiro-Wilk test. Normally distributed data were presented as mean ± standard deviation (SD) and frequency (%). Parametric data underwent Student's *t*-test and Chi-squared test to compare the significant difference between the two groups. Whereas, quantitative non-parametric measurements were presented as interquartile ranges (IQR) and compared with the non-parametric Mann-Whitney U test if the data failed to follow a normal distribution or homoscedasticity. The α-diversity was evaluated by Chao1, Shannon, PD whole tree, and Simpson indices, and the β-diversity was assessed with unweighted and weighted ANOSIMs Unifrac analysis. Principal coordinate analysis was carried out for β-diversity. The Wilcoxon test was conducted to analyze gut microbiota differences between the two groups. The linear discriminant analysis effect size (LEfSe) was employed to reveal the unique bacterial signatures identified in DCI and T2DM patients. In order to estimate the relations of altered gut microbiota with the DCI clinical characteristics, SCFAs, and inflammatory cytokines, Spearman's rank correlation coefficient test was applied with the R package “cor. test.” *P* < 0.05 was considered statistically significant in the analyses.

## Results

### Demographic information and clinical parameters of DCI and T2DM groups

A total of 60 T2DM patients were enrolled in this study, of whom 30 were diagnosed with MCI (DCI group, 68.67 ± 6.44 years old, 63% female) and 30 were without MCI (T2DM group, 69.73 ± 5.11 years old, 43% female). Significant differences in MoCA, MMSE, and CDT scores were found between DCI and T2DM groups (*P* < 0.001). No significant differences were observed in age, gender, education level, BMI, WC, history of hypertension, exercise frequency, smoking, and alcohol consumption between the two groups (Table 1). Moreover, no significant differences existed in the levels of FBG, HbA_1C_, ALT, AST, TBIL, CR, BUN, TC, TG, LDL-C, and HDL-C between the two groups (Table 2).

**Table 1:** The demographic characteristics and comprehensive assessment of DCI and T2DM participants.

**Variables**	**DCI group (*****N*** **= 30)**	**T2DM group (*****N*** **= 30)**	***P-*** **value**
Demographic variables			
Age, years	67 (65, 71)	70.00 (66, 72)	0.235
Women, n (%)	19 (63)	13 (43)	0.121
Education^a^, n (%)			
Illiteracy and primary school	18 (62)	16 (62)	0.249
Secondary school	9 (31)	10 (38)	
Technical diploma or above	2 (7)	0 (0)	
BMI, kg/m^2^	27.50 ± 4.59	27.70 ± 3.39	0.849
Waist circumference, cm	93.30 ± 8.40	92.50 ± 7.71	0.702
History of hypertension, n (%)			
Yes	28 (93)	25 (83)	0.421
No	2 (7)	5 (17)	
Comprehensive assessment			
Exercise frequency, n (%)			
Every day	6 (20)	10 (33)	0.106
Occasionally	3 (10)	7 (23)	
Never	21 (70)	13 (43)	
Smoking (still smoking), n (%)	5 (17)	6 (20)	0.739
Alcohol consumption, n (%)	5 (17)	12 (40)	0.101
Never	25 (83)	18 (60)	
Occasionally	3 (10)	5 (17)	
Frequently	2 (7)	7 (23)	
MoCA	14.50 (11.00, 17.25)	25.00 (22.75,26.25)	<0.001
MMSE	23 (19.00, 25.00)	27 (25.00, 29.00)	<0.001
CDT	0 (0.00, 2,25)	3 (2.00, 3.00)	<0.001

a *: missing 5; b: missing 5; c: missing 4; d: missing 4; e: missing 5; MMSE, Mini-Mental State Examination; MoCA, Montreal Cognitive Assessment; CDT, Clock Drawing Test*.

**Table 2:** The hematological parameters of DCI and T2DM participants.

**Hematological parameters**	**DCI group (*****N*** **= 30)**	**T2DM group (*****N*** **= 30)**	***P-*** **value**
FBG	7.80 (7.28, 9.02)	7.75 (7.25, 7.90)	0.247
HbA_1C_	7.00 (6.70, 7.40)	6.5 (6.50, 7.43)	0.085
ALT	13.00 (10.00, 17.25)	14.00 (10.00, 17.50)	0.818
AST	19.50 (16.00, 22.25)	19.00 (18.00, 21.25)	0.853
TBIL	16.27 ± 5.39	17.93 ± 6.60	0.288
Cr	61.50 (54.00, 78.00)	70.50 (57.25, 79.00)	0.584
BUN	4.70 (4.25, 5.78)	5.40 (4.08, 6.03)	0.684
TC	4.48 ± 1.01	4.53 ± 0.58	0.840
TG	1.29 (0.93, 3.09)	1.33 (0.86, 1.92)	0.355
LDL-C	2.65 ± 0.88	2.94 ± 0.51	0.128
HDL-C	1.24 ± 0.39	1.32 ± 0.36	0.414

*FBG, fasting blood glucose; HbA_1C_, glycated hemoglobin A1c; ALT, alanine aminotransferase; AST, aspartate aminotransferase; TBIL, total bilirubin; Cr, creatinine; BUN, blood urea nitrogen; TC, total cholesterol; TG, triglyceride; LDL-C, low-density lipoprotein cholesterol; HDL-C, high-density lipoprotein cholesterol*.

### Alpha and beta diversity between DCI and T2DM groups

According to a Venn diagram, except for the overlapped 512 OTUs, 63 and 40 OTUs were discovered in DCI and T2DM groups, respectively (Supplementary Figure 1A). In addition, species accumulation curves were plotted to describe the increase in species with increasing sampling size (Supplementary Figure 1B). The rarefaction curves of richness were plotted, showing a reasonable amount of sequencing data and a sufficient sequencing depth (Supplementary Figure 1C).

**Figure 1:**
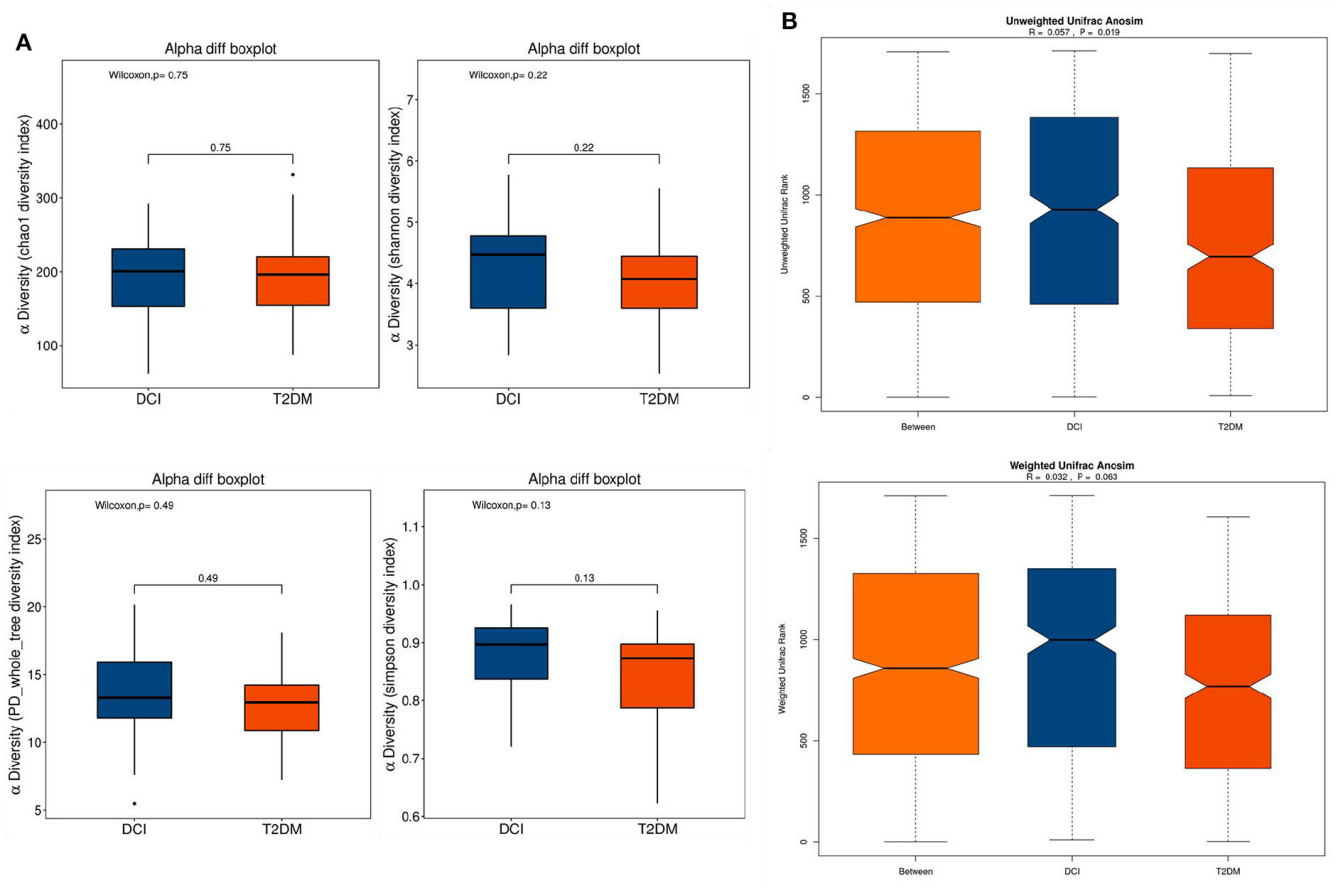
The α-diversity and β-diversity indices of the fecal microbiota in DCI and T2DM groups. **(A)** Box plots depict differences in the fecal microbiota diversity indices between the two groups according to the Chao1, Shannon, PD whole tree and Simpson indices based on the OTU counts. Each box plot represents the median, interquartile range, minimum, and maximum values. **(B)** Unweighted and weighted ANOSIM Unifrac analysis based on the distance matrix of UniFrac dissimilarity of the fecal microbial communities in DCI and T2DM groups. Respective ANOSIM R values and significant *P*-values show the community variation between two groups. The axes represent the two dimensions explaining the greatest proportion proportion of variance in the communities. OTU, operational taxonomic unit, ANOSIM, analyses of similarities.

The α-diversity and β-diversity were assessed to determine the difference in overall gut microbiota diversity between DCI and T2DM groups. The α-diversity indexes (including Chao 1, Shannon, PD whole tree, and Simpson indices) were employed to characterize community richness and diversity. Based on these indices, no significant difference appeared between DCI and T2DM groups (Figure 1A). Then, a significant discrepancy in β-diversity was identified based on the unweighted UniFrac ANOSIM metric (qualitative, ANOSIM *R* = 0.057, *P* = 0.019), but not the weighted UniFrac ANOSIM metric (quantitative, ANOSIM *R* = 0.032, *P* = 0.063) between the two groups (Figure 1B).

### Changes in gut microbiota composition between DCI and T2DM groups

**Figure 2:**
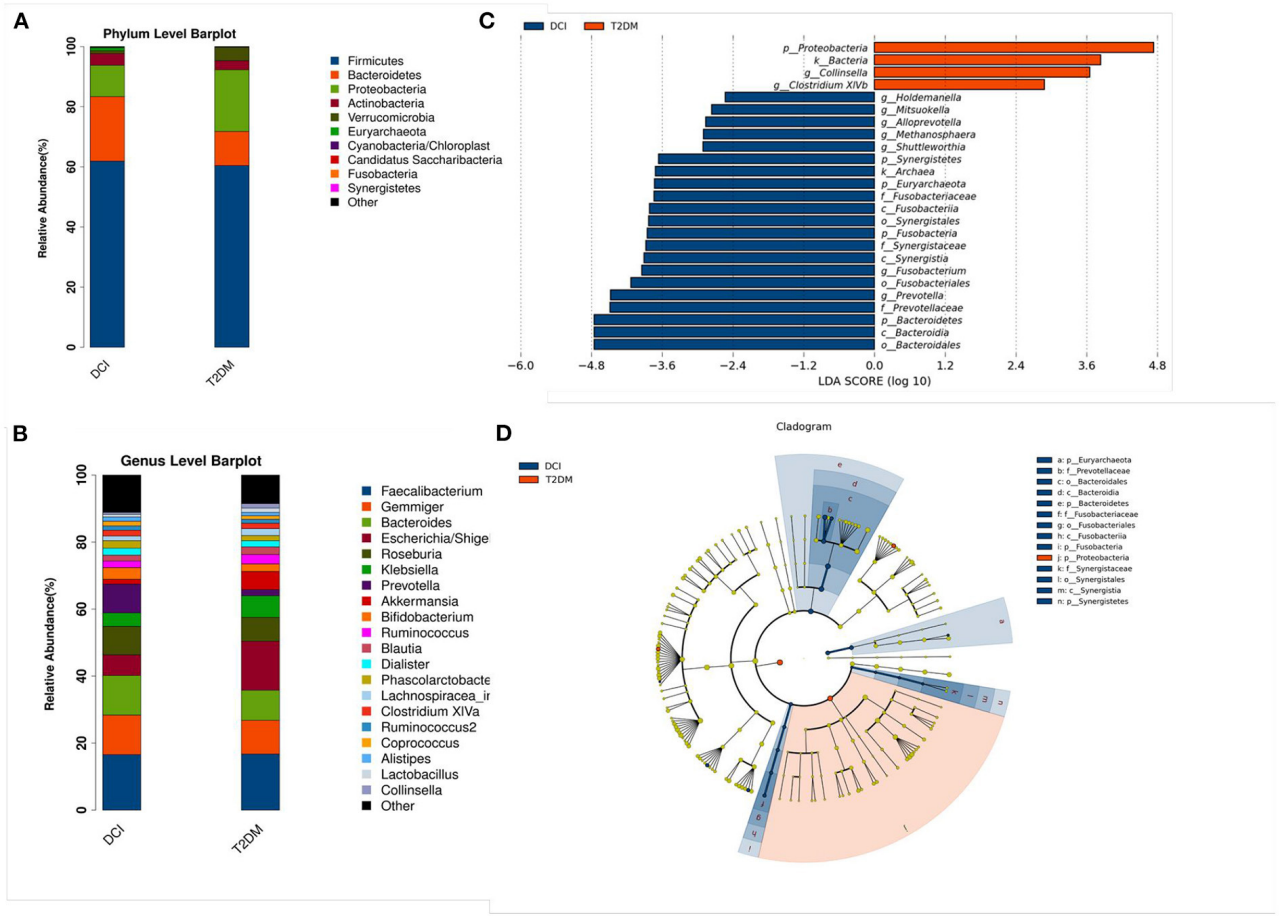
Changes of gut microbiota between DCI and T2DM groups at the phylum and genus level. **(A,B)** The bar chart of gut microbiota composition at the level of phylum **(A)** and genus **(B)** between the two groups. **(C,D)** Linear discriminant analysis (LDA) effect size (Lefse) plot and cladogram revealed the unique bacterial signatures identified in DCI and T2DM patients. The LDA scores (log10) > 2 and *P* < 0.05 are listed.

As shown in Figure 2, the relative abundance of numerous taxa between DCI and T2DM patients was revealed by bacterial taxonomy and the OTU abundance at the phylum and genus levels. The dominant five phyla of gut microbiota in DCI and T2DM groups were *Firmicutes, Bacteroidetes, Proteobacteria, Actinobacteria*, and *Verrucomicrobia*. Compared to T2DM patients, DCI patients represented a greater abundance of the phyla *Firmicutes, Bacteroidetes*, and *Actinobacteria* but a lower abundance of *Proteobacteria* and *Verrucomicrobia* (Figure 2A). At the genus level, DCI patients showed a higher abundance of the genera *Gemmiger, Bacteroides, Roseburia, Prevotella*, and *Bifidobacterium* but a lower abundance of *Escherichia* and *Akkermansia* than T2DM patients (Figure 2B).

LEfSe analysis has been generally used for a supervised comparison of the gut microbiota among different groups (41, 42). In this study, a logarithmic LDA score cutoff of 2.0 was set to identify significant taxonomic differences between DCI and T2DM groups. The findings revealed a significant difference in the gut microbiota of DCI and T2DM groups. At the genus level, the relative abundance of *Holdemanella, Mitsuokella, Alloprevotella, Methanosphaera, Shuttleworthia, Fusobacterium*, and *Prevotella* were higher in DCI group, whereas that of *Collinsella* and *Clostridium XlVb* were greater in T2DM group (Figures 2C,D).

### Plasma SCFAs and inflammatory cytokines in DCI and T2DM groups

**Figure 3:**
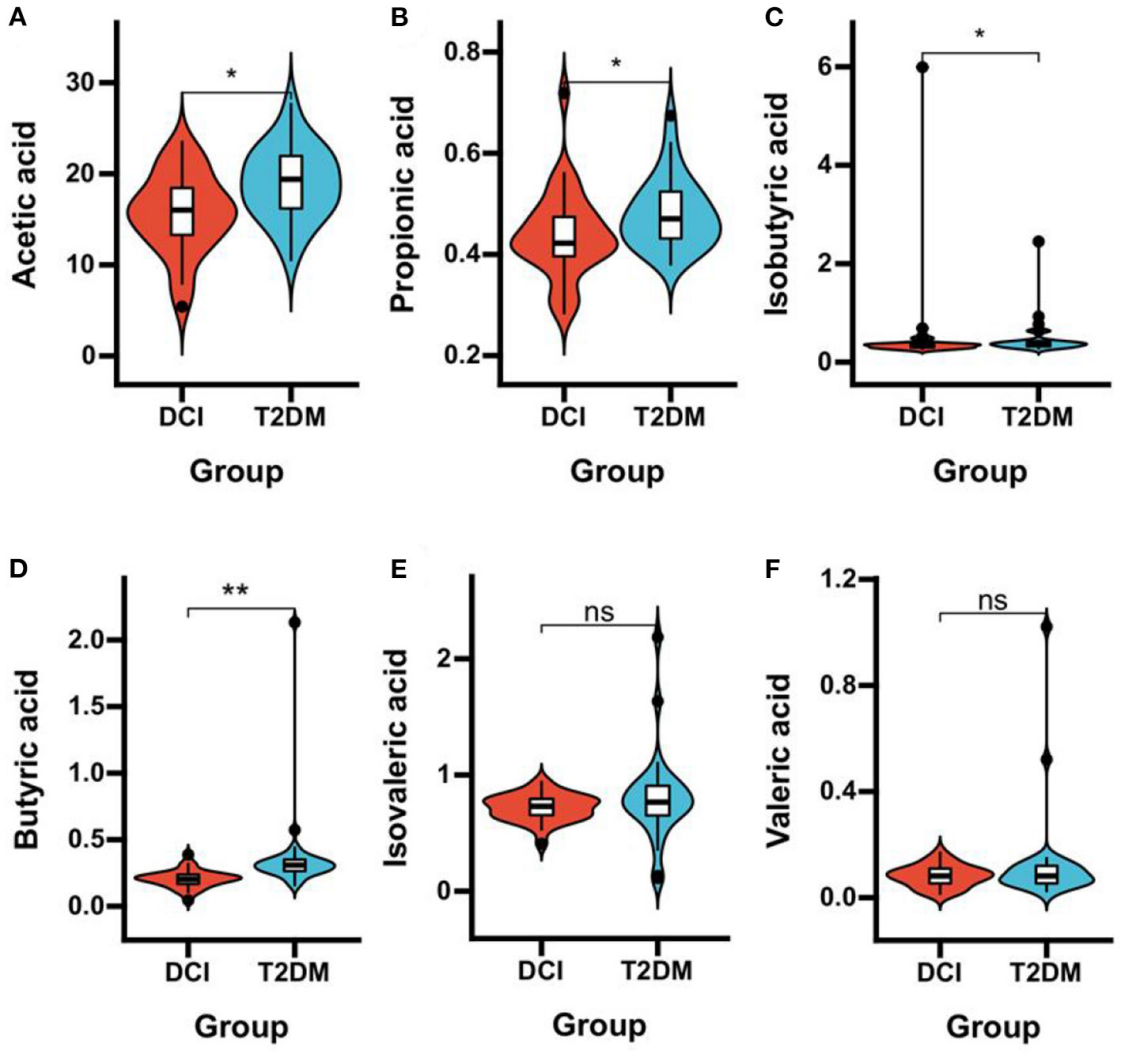
Comparison of the plasma concentrations of SCFAs in DCI and T2DM groups. Violin plot showing data density and median with interquartile range for plasma concentrations of acetic acid **(A)**, propionic acid **(B)**, isobutyric acid **(C)**, butyric acid **(D)**, isovaleric acid **(E)**, and valeric acid **(F)** in DCI and T2DM groups. *P < 0.05, **P < 0.001.

**Figure 4:**
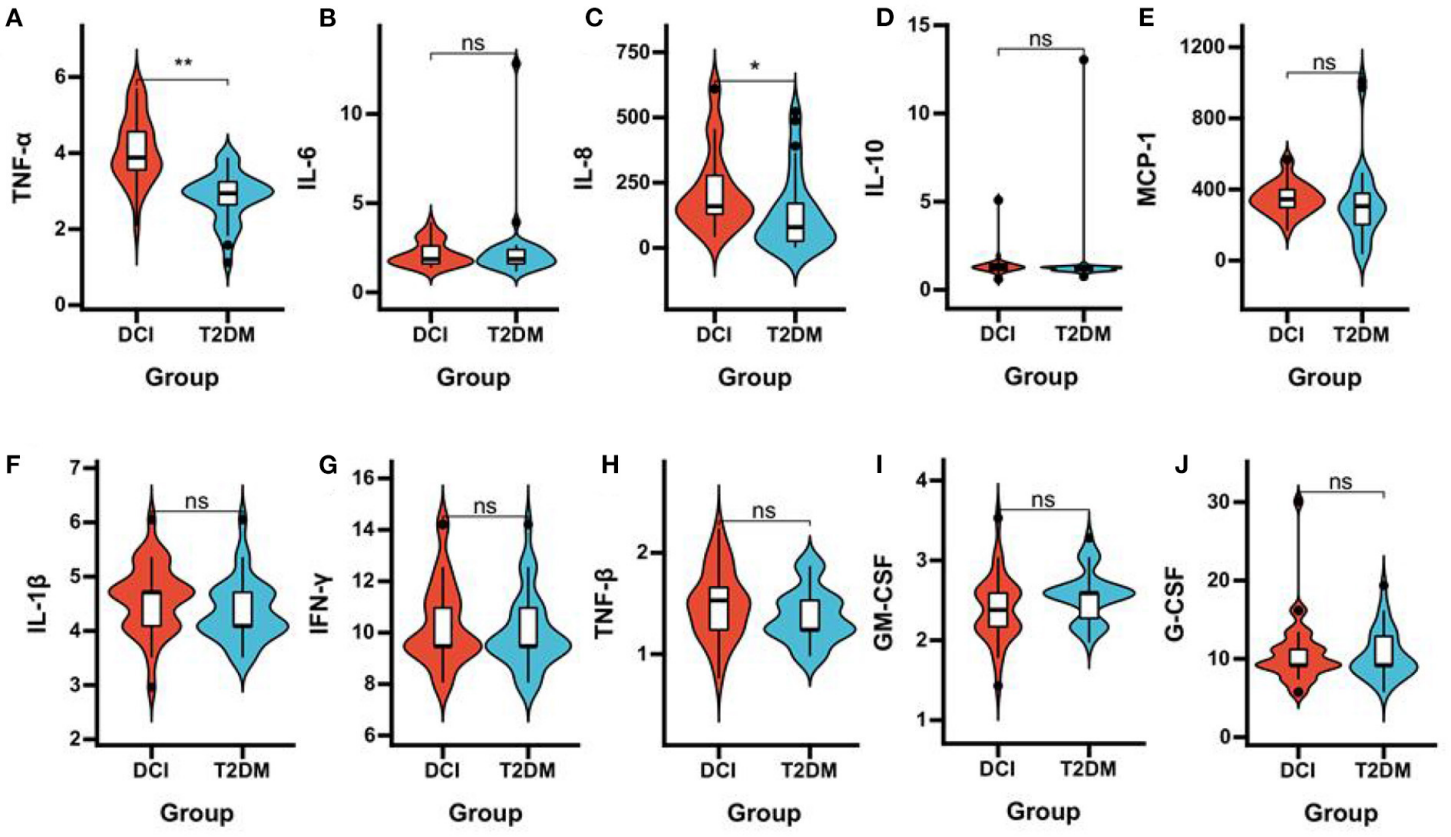
Comparison of the plasma concentrations of inflammatory cytokines in DCI and T2DM groups. Violin plot showing data density and median with interquartile range for plasma concentrations of TNF-α **(A)**, IL-6 **(B)**, IL-8 **(C)**, IL-10 **(D)**, MCP-1 **(E)**, IL-1β **(F)**, IFN-γ **(G)**, TNF-β **(H)**, GM-CSF **(I)**, G-CSF **(J)** in DCI and T2DM groups.*P < 0.05, **P < 0.001.

DCI patients represented significantly poorer plasma concentrations of acetic acid (*P* = 0.006), propionic acid (*P* = 0.033), isobutyric acid (*P* = 0.047), and butyric acid (*P* < 0.001) than T2DM patients (Table 3 and Figures 3A–F). The levels of TNF-α (*P* < 0.001) and IL-8 (*P* = 0.005) in plasma were significantly greater in DCI patients than in T2DM patients. The MCP-1, IL-1β, and TNF-β levels were higher in DCI group than in T2DM group, with no significant difference (*P* > 0.05) (Table 3 and Figures 4A–J).

**Table 3:** The plasma concentrations of SCFAs and inflammatory cytokines in DCI and T2DM groups.

	**DCI group (*****N*** **= 30)**	**T2DM group (*****N*** **= 30)**	***P-*** **value**
SCFAs			
Acetic acid, μg/mL	16.01 (12.99, 18.52)	19.40 (16.08, 22.07)	0.006
Propionic acid, μg/mL	0.42 (0.39, 0.48)	0.47 (0.43, 0.53)	0.033
Isobutyric acid, μg/mL	0.35 (0.30, 0.37)	0.37 (0.31, 0.43)	0.047
Butyric acid, μg/mL	0.20 (0.17, 0.24)	0.31 (0.26, 0.35)	<0.001
Isovaleric acid, μg/mL	0.73 (0.65, 0.80)	0.77 (0.64, 0.91)	0.214
Valeric acid, μg/mL	0.08 (0.05, 0.11)	0.08 (0.05, 0.12)	0.563
Inflammatory cytokines			
TNF-α, pg/ml	3.88 (3.56, 4.56)	2.94 (2.57, 3.24)	<0.001
IL-6, pg/ml	1.86 (1.61, 2.74)	1.86 (1.61, 2.39)	0.676
IL-8, pg/ml	159.77 (124.66, 287.60)	79.31 (22.33, 171.84)	0.005
IL-10, pg/ml	1.28 (1.10, 1.48)	1.28 (1.10, 1.28)	0.071
MCP-1, pg/ml	344.64 (296.58, 407.53)	305.61 (197.29, 400.46)	0.089
IL-1β, pg/ml	4.71 (4.09, 4.79)	4.09 (4.09, 4.71)	0.090
IFN-γ, pg/ml	9.47 (9.47, 11.16)	9.47 (9.47, 10.96)	0.474
TNF-β, pg/ml	1.53 (1.24, 1.74)	1.24 (1.24, 1.53)	0.140
GM-CSF, pg/ml	2.38 (2.17, 2.59)	2.59 (2.17, 2.59)	0.083
G-CSF, pg/ml	9.18 (9.18, 11.47)	9.18 (8.95, 13.43)	0.909

### Gut microbiota species correlated with DCI clinical characteristics

**Figure 5:**
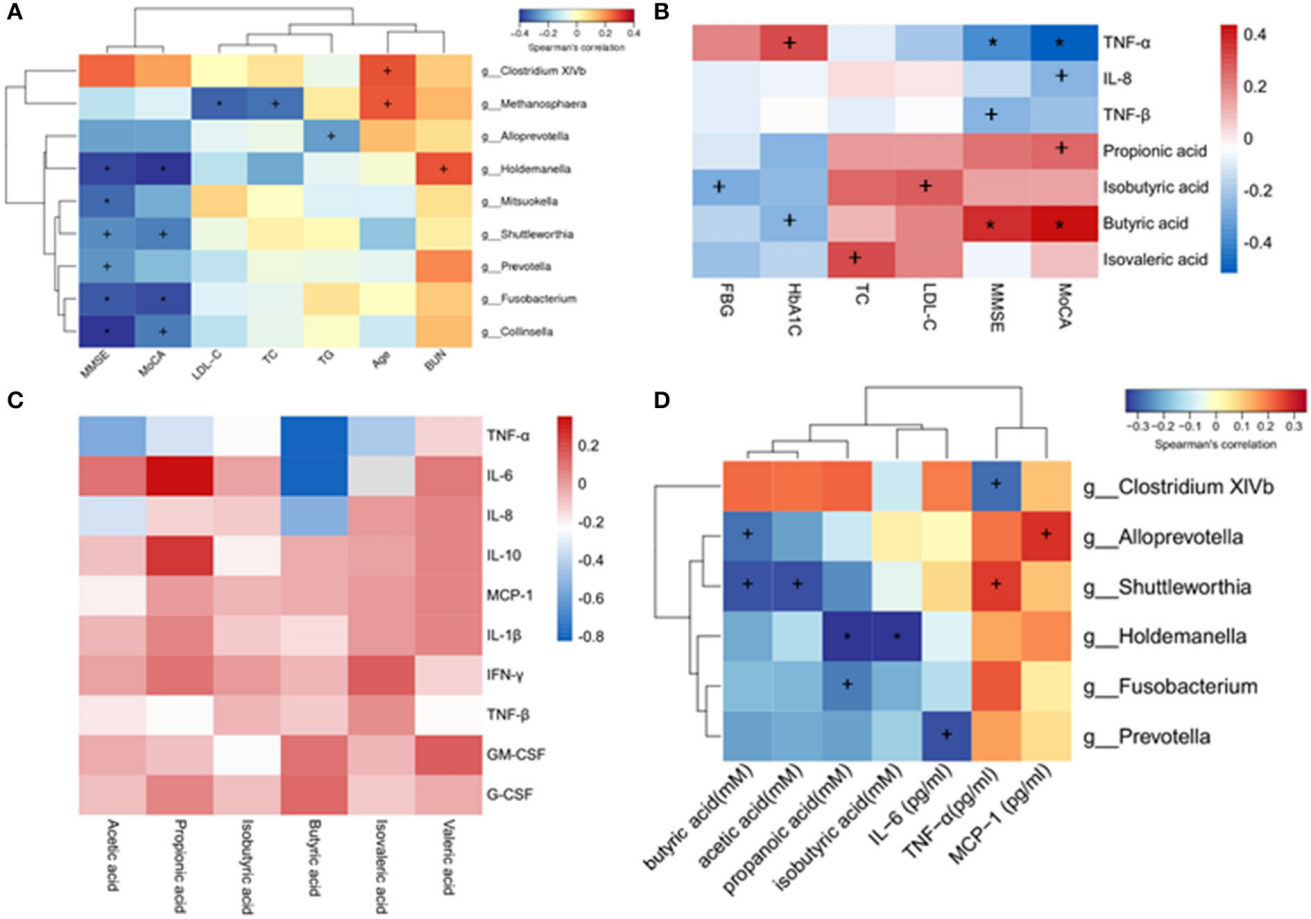
Heatmaps showing correlations between altered gut microbiota (genus level) and DCI clinical characteristics. **(A)** Correlations between altered gut microbiota and DCI clinical characteristics. **(B)** Correlations between plasma levels of SCFAs, inflammatory cytokines and DCI clinical characteristics. **(C)** Correlations between plasma levels of SCFAs and inflammatory cytokines. **(D)** Correlations between altered gut microbiota and plasma levels of SCFAs and inflammatory cytokines. ^+^*P* < 0.05; *P < 0.01.

A correlation analysis was performed to detect the relations between gut microbiota and DCI clinical characteristics. The results indicated that the genera *Holdemanella, Mitsuokella, Shuttleworthia, Prevotella, Fusobacterium*, and *Collinsella* were negatively correlated with the MMSE scores. In addition, the genera *Holdemanella, Shuttleworthia, Fusobacterium*, and *Collinsella* were negatively correlated with the MoCA scores. The genera *Methanosphaera* was negatively correlated with LDL–C and TC, and the genera *Alloprevotella* was negatively correlated with TG. The genera *Holdemanella* was positively correlated with BUN. In addition, the genera *Clostridium XlVb* and *Methanosphaera* were positively correlated with age (Figure 5A).

### Plasma SCFAs and inflammatory cytokines correlated with DCI clinical characteristics

A negative correlation was found between the plasma levels of TNF-α and MMSE, MoCA. Plasma IL-8 was negatively correlated with MoCA scores, while TNF-β was negatively correlated with MMSE scores. Regarding SCFAs, propionic acid was positively correlated with MoCA scores, whereas butyric acid was positively correlated with MMSE and MoCA scores. A negative correlation was observed between the isobutyric acid and FBG (Figure 5B).

### Plasma SCFAs correlated with inflammatory cytokines

Spearman's rank correlation coefficient test was utilized to evaluate the SCFA-inflammatory cytokine relationship based on the above data. TNF-α was negatively correlated with the concentrations of acetic acid, propionic acid, butyric acid, and isovaleric acid in plasma. The plasma concentration of propionic acid was positively correlated with IL-6 and IL-10 (Figure 5C), while that of acetic acid and butyric acid was negatively correlated with IL-8.

### Gut microbiota correlated with plasma SCFAs and inflammatory cytokines

The correlations between gut microbiota abundance (genus level) and plasma SCFA concentration or inflammatory cytokines were further investigated. Interestingly, most of the identified genera of gut microbiota were negatively correlated with SCFAs. Specifically, the genera *Alloprevotella* and *Shuttleworthia* were negatively correlated with butyric acid, and *Shuttleworthia* was also negatively correlated with acetic acid. The genera *Holdemanella* was negatively correlated with propanoic acid and isobutyric acid, and *Fusobacterium* was negatively correlated with propanoic acid. Concerning plasma inflammatory cytokines, it was found that the genera *Clostridium XlVb* was negatively correlated with TNF-α, and *Prevotella* was negatively correlated with IL-6. The genera *Alloprevotella* was positively correlated with MCP-1, and *Shuttleworthia* was positively correlated with TNF-α (Figure 5D).

## Discussion

This study used 16S rRNA gene sequencing to investigate the gut microbiota composition in DCI patients. The results showed a significant qualitative difference in the β-diversity index of DCI and T2DM patients. However, no statistically significant differences were observed between the two groups in α-diversity indices, indicating the alteration of gut microbiota composition in DCI patients. Previous research conducted in Wuxi (a city in Southern China) revealed no difference existed in the fecal α-diversity and β-diversity indexes in T2DM patients with or without impaired cognition (15). Due to the fact that diet markedly influences the composition of the gut microbiota (43, 44). The present research was conducted in Tianjin city in Northern China. The dietary patterns of inhabitants in Northern and Southern China vary significantly, which explains the inconsistent results of α- and β-diversity in these two studies.

Subsequently, the gut microbiota composition was examined by observing the bacterial species at the phylum and genus levels. It was found that DCI patients showed a higher abundance of *Firmicutes, Bacteroidetes*, and *Actinobacteria* than T2DM patients. Increased *Firmicutes* were generally associated with dysbiotic microbiome signatures and poor health outcomes, implying the potential dysbiotic gut microbiota in DCI patients (45). Moreover, there are no previous studies linked the *Bacteroidetes* to DCI patients. But, previous studies explored *Bacteroidetes* in patients with AD, MCI, and Parkinson's disease (PD), identifying a lessened abundance of *Bacteroidetes* in patients with AD/PD (42, 45). Nevertheless, Vogt and colleagues (46) found that the relative abundance of *Bacteroidetes* was enhanced in AD patients. This difference may be due to variation in the geographical background, dietary habits, and age of subjects. The latest study found for the first time that Dendrobium mixture could regulate phyla of *Firmicutes* and *Bacteroidetes* to restore cognitive and memory function in DCI mice (27). Consistent with the present study, previous studies indicated that *Actinobacteria* was elevated at the class level in T2DM patients with cognitive impairment (15). It should be noted that the abundance of the phyla *Proteobacteria* and *Verrucomicrobia* was much lower in DCI group than in T2DM group, indicating these phyla might be associated with cognitive impairment in T2DM patients. *Proteobacteria* and *Verrucomicrobia* were reported to play a crucial role in AD (47, 48). An animal study has identified that the content of *Proteobacteria* and *Verrucomicrobia* plunged in the gut of APP/PS1 mice compared to that of wild-type animals (49). The only representative human gut microbiota capable of being cultured in *Verrucomicrobia* was Akk (17), which was found to exhibit a lower abundance at the genus level in DCI group in this study.

According to the analysis of the differences in gut microbiota between the two groups at the genus level, a lower abundance of the genus *Escherichia* was observed in DCI group than in T2DM group, inconsistent with those reports of a high abundance of *Escherichia* in both fecal and blood samples from MCI and AD patients (23). Gram-negative *Escherichia* might be a risk factor for MCI and AD because it could express and secrete Aβ proteins (50). Additionally, *Escherichia* endotoxin promoted the formation of Aβ proteins *in vitro*, facilitating AD pathogenesis. To our knowledge, no research on *Escherichia* in DCI patients has been reported. Therefore, further research is needed to examine the association between *Escherichia* and the cognition of DCI patients.

Numerous studies have revealed the impact of SCFAs (acetic acid, propionic acid, and butyric acid) on human metabolism. SCFAs are predominantly produced in the colon, of which only a small percentage enter the peripheral circulation after being absorbed and used by colonocytes (51), resulting in a low SCFA level in the peripheral circulation (52). Acetic acid is the most abundant product of bacteria in the colon, whereas propionic acid and butyric acid are produced in smaller quantities. In this study, the concentrations of acetic acid, propionic acid, isobutyric acid, and butyric acid in plasma were significantly reduced in DCI patients compared to T2DM patients. A recent animal study discovered the protective role of microbiota metabolite acetic acid in cognitive functions and that long-term acetic acid deficiency is a risk factor for cognitive decline (33). Acetic acid could be produced by Akk (53) and our study showed that the abundance of Akk was lower in DCI patients than in T2DM patients. These results indicated that Akk might play an important role in the DCI pathology by regulating the acetic acid level, but the corresponding underlying mechanism is still unknown. *Fusobacterium* and *Shuttleworthia* are *two* genera producing butyric acid (54). Interestingly, LEfSe analysis showed a greater relative abundance of the two genera in DCI than in T2DM patients in our study, suggesting that the gut microbiota and SCFAs in DCI patients might be complicated, and the SCFA level was also affected by factors such as inflammation. In addition, the reason might be that the healthy older participants without T2DM and cognitive impairment were not enrolled as a control group. Therefore, the gut microbiota and SCFA levels of DCI patients are unclear compared to those of healthy older adults.

Meta-analyses revealed that a dietary supply of acetic acid significantly reduced FBG in T2DM (55). Moreover, acetic acid presented anti-inflammatory effects and acted as a histone deacetylase (HDAC) 1 inhibitor, inhibiting microglial activation (56). In addition, Bartolomaeus et al. (57) found that propionic acid treatment could reduce systemic inflammation in ApoE^−/−^mice. But propionic acid was also discovered to disrupt the production of neurotransmitters and activate microglia. SCFAs have both pro- and anti-inflammatory activities, and dietary supplementation shows different effects on inflammation because it appears to be strongly influenced by SCFA concentration (51). Therefore, the role of propionic acid in DCI requires further investigation. Butyric acid represents therapeutic benefits for AD *via* epigenetic mechanisms by inhibiting HDAC, normalizing aberrant histone acetylation, and increasing BDNF expression. Sun et al. (34) performed FMT to alleviate AD-like pathogenesis in APP/PS1 transgenic mice, from which they found an increased butyric acid level. Such being the case, no articles have been published on SCFA changes in DCI patients to date. PD patients represented a higher plasma butyric acid level but a significantly lower level of fecal butyric acid (51). Reduced fecal butyric acid may be associated with cognitive decline in PD patients. Considering the minute changes in plasma SCFAs and the small sample size in this study, the findings need further verification through a larger sample size study. The present study revealed that the concentrations of propionic acid and butyric acid were positively associated with MoCA scores, and the butyric acid level was positively correlated with MMSE scores, suggesting that low levels of propionic acid and butyric acid might worsen the cognitive function of DCI patients.

TNF-α is a pro-inflammatory cytokine, of which an elevated level has been found in various diseases (24), including DCI (21). In the present study, the plasma TNF-α concentration was significantly higher in DCI patients than in T2DM patients. TNF-α and its receptors, i.e., soluble tumor necrosis factor receptors (sTNFR) 1 and 2 capable of regulating numerous physiological processes in CNS, have been reported to aggravate the Aβ and tau pathologies in AD patients (58). In addition, a higher level of IL-8 was observed in DCI patients than T2DM patients. IL-8 is secreted by activated neutrophils and plays a role in neutrophil trafficking and activation, which can cause neurotoxicity and neuronal cell death *in vitro*, while the IL-8 receptor antagonist shows neuroprotective benefits in the AD mouse model (59). A longitudinal observational study also identified that the IL-8 level predicted the degeneration of executive function in AD patients (60). The levels of TNF-α and IL-8 in plasma were negatively correlated with MoCA scores, indicating a high level might contribute to DCI development.

SCFAs can activate the MAPK pathway through the G protein-coupled receptor and inhibit the β-arrestin2/NF-kB pathway and the synthesis of cyclic adenosine monophosphate (cAMP), thereby regulating inflammation and immunity-associated gene expression in macrophages, neutrophils, and dendritic cells (61). The correlation of SCFAs with inflammatory cytokines in DCI patients was identified by Spearman's rank correlation coefficient test, and the results showed that the plasma concentrations of acetic acid, propionic acid, butyric acid, and isovaleric acid were negatively correlated with TNF-α. Acetic acid and butyric acid were negatively correlated with IL-8. These results indicated that a higher SCFA level was correlated with a lower-level inflammation. The anti-inflammatory properties of acetic acid, propionic acid, and butyric acid have been found in the periphery (62). Therefore, the findings in this study support the beneficial effects of SCFAs. Despite this, SCFAs might promote neuroinflammation when the concentration exceeds a range in mice (63).

Gut microbiota was correlated with DCI clinical characteristics in the present study, including MMSE, MoCA, LDL–C, TC, TG, age, and BUN. The genera *Holdemanella, Mitsuokella, Shuttleworthia, Prevotella, Fusobacterium*, and *Collinsella* were negatively correlated with the MMSE scores, while *Holdemanella, Shuttleworthia, Fusobacterium*, and *Collinsella* were negatively correlated with the MoCA scores. This indicated that the increased abundance of these gut microbiota might impair the cognitive function of DCI patients. The genera *Methanosphaera* was negatively correlated with LDL–C and TC, while the genus *Alloprevotella* was negatively correlated with TG, suggesting that certain bacteria might play a role in lipid metabolism.

In addition, the correlation between gut microbiota and SCFAs or inflammatory cytokines was confirmed in DCI patients. In this study, the genera *Alloprevotella* was negatively correlated with butyric acid, indicating a high abundance of *Alloprevotella* in DCI patients was associated with a low level of butyric acid. Therefore, the level of the circulating butyric acid could be increased by regulating the *Alloprevotella* level. The genera *Holdemanella* was negatively correlated with propanoic acid and isobutyric acid, while *Fusobacterium* was negatively correlated with propanoic acid, suggesting that these genera might bring about a unique breakthrough in understanding the generation and regulation of SCFAs in DCI progression. It is found that the genera *Clostridium XlVb* was negatively correlated with TNF-α, whereas *Shuttleworthia* was positively correlated with TNF-α, and both genera belonged to the *Candidatus Saccharibacteria* (hereafter referred to as *Saccharibacteria*) phylum, which is a well-described candidate phylum not be successfully isolated. They have been detected in a variety of natural environments, but little is known about their phylogeny and physiology, particularly those linked to human diseases (64). Although *Saccharibacteria* has been linked to periodontitis (65), no studies have revealed the dysregulation of *Saccharibacteria* in DCI patients. The genera *Prevotella* was negatively correlated with IL-6. *Prevotella* species are Gram-negative anaerobic bacteria of the *Bacteroidetes* phylum and are commonly referred to as commensal bacteria because of their widespread presence in the healthy human body and infrequent involvement in infections. However, emerging studies have indicated the relationship between a growing *Prevotella* abundance and inflammatory disorders. For example, in patients with periodontitis, *Prevotella* represented an enhanced ability to induce inflammatory mediators (IL-6, IL-8, and TNF-α) (66). In contrast, no study was searched to link *Prevotella* to IL-6 in DCI patients. Therefore, the causal and potential pathogenic roles of *Prevotella* and inflammatory markers in DCI patients remain to be ascertained.

The limitations of this study should be considered. First, the participants were recruited from a single geographic area and had a small sample size, which may limit the generalization or extrapolation of the findings. Studies with different populations and larger samples are required to confirm the findings of the study in the future. Second, healthy older participants without T2DM and cognitive impairment were not enrolled as a control group, which might prohibit the observation of the gut microbiota-T2DM relations. Third, the present study is a cross-sectional study, and thus the causality between the gut microbiota changes and DCI is still unclear. For this reason, a longitudinal study focusing on cognitive decline in T2DM patients is in urgent demand to validate the findings of this study. In addition, the germ-free mouse model should be established for FMT so as to further identify the causal relationship between the gut microbiota and DCI. Fourth, although it is known that anti-diabetic medication can affect gut microbiota and plasma concentrations of butyrate, it is yet to be known whether anti-diabetic medication has any influence on gut microbiota in our study. Finally, shotgun metagenomics analysis can be performed to provide more details about the gut microbiota in DCI patients.

## Conclusions

This paper demonstrated that alterations in gut microbiota were accompanied by changes in the levels of SCFAs and inflammatory cytokines in DCI patients, which might pose threats to DCI development. The present study lays a foundation for improving the understanding of the relationship between gut microbiota, inflammatory cytokines, and host metabolism. These findings suggest that novel microbiota-based therapy is a promising target for DCI treatment in the future.

## Data availability statement

The raw data supporting the conclusions of this article will be made available by the authors, without undue reservation.

## Author contributions

YD wrote the initial draft. YD and XL contributed to study conceptualization, design, manuscript review, and approval. YA and YS contributed to survey implementation. YA and XL contributed to data analysis and interpretation. YL reviewed the initial manuscript. All authors revised and approved the final version.

## Funding

This research was funded by National Natural Science Foundation of China (82003456), and the Fundamental Research Funds for the Central Universities.

## Conflict of interest

The authors declare that the research was conducted in the absence of any commercial or financial relationships that could be construed as a potential conflict of interest.

## Publisher's Note

All claims expressed in this article are solely those of the authors and do not necessarily represent those of their affiliated organizations, or those of the publisher, the editors and the reviewers. Any product that may be evaluated in this article, or claim that may be made by its manufacturer, is not guaranteed or endorsed by the publisher.
