# Supplementary material for: Association of gut microbiota with sort-chain fatty acids and inflammatory cytokines in diabetic patients with cognitive impairment: A cross-sectional, non-controlled study
Source: Front Nutr. 2022 Jul 22;9:930626. doi: 10.3389/fnut.2022.930626 (PMC9355148; doi:10.3389/fnut.2022.930626)
Supplement: Supplementary file 2 [file Image_1.pdf]

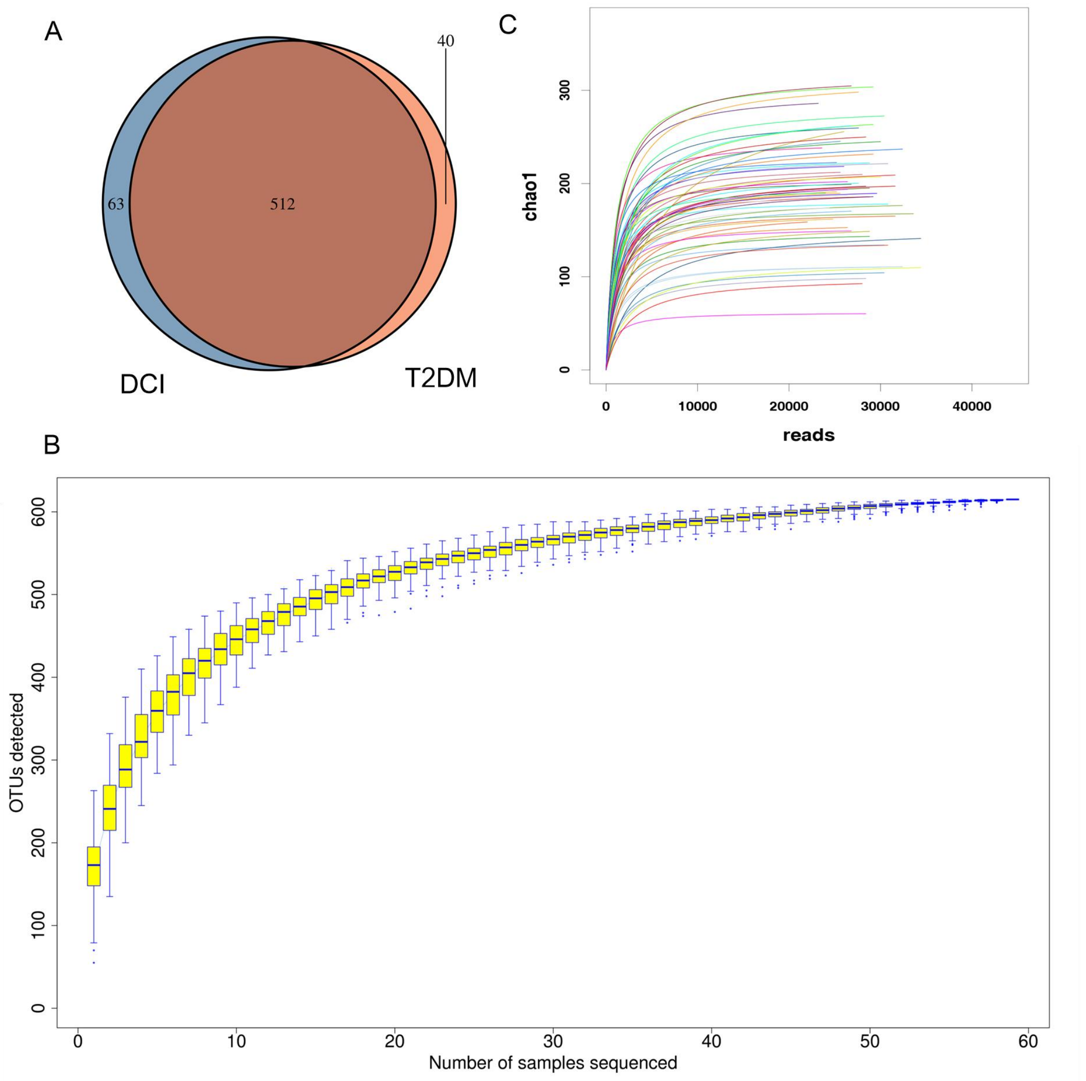

FIGURE S1

The α-diversity and β-diversity of the fecal microbiome alteration in DCI and T2DM groups. **(A)** Venn diagram in DCI and T2DM groups. **(B)** Species accumulation curve of Specaccum showed the adequate sampling and high species richness. **(C)** Rarefaction curves based on the Chao 1 index showed the reasonable sequencing data and the sufficient sequencing depth.
